# Ocular Vestibular Evoked Myogenic Potential Using Different Test Stimuli

**DOI:** 10.1155/2013/161937

**Published:** 2013-08-06

**Authors:** Dessai Teja Deepak, Jayashree S. Bhat, Kaushlendra Kumar

**Affiliations:** Department of Audiology & Speech Language Pathology, Kasturba Medical College, Manipal University, Mangalore 575 001, India

## Abstract

*Aim*. Ocular Evoked Myogenic Potential (oVEMP) are short latency potentials evoked by higher acoustic stimulation. In this study, we aimed at comparing the click, 500 Hz mixed modulated, and 500 Hz short duration tone burst stimuli using oVEMP. *Material*. Click, 500 Hz mixed modulated and 500 Hz short duration tone burst stimuli were used for the study. *Method*. Conventional sampling and conveneint study design were used. Sixty healthy subjects underwent contralateral oVEMP testing maintaining 30 degrees upward gaze. Single channel electrode montage was applied to record oVEMP response. *Results*. On statistical analysis the three stimuli evoked equal response rates (100%), and when latency of n1 and p1 and peak-peak amplitude were compared, the click evoked showed significantly early latency and lower peak-peak amplitude than the 500 Hz stimuli. Five hundred Hz stimuli did not show significant difference in latency and peak-peak amplitude of n1-p1. *Discussion*. Thus, 500 Hz stimuli can evoke better latency and peak-peak amplitude. oVEMP has good clinical significance in diagnosing subjects with vestibular dysfunction. To add to the sensitivity of the oVEMP test, 500 Hz stimuli may also be used as it can evoke better oVEMP responses in clinical population with good morphology.

## 1. Introduction

Human auditory system consists of otolith, namely, saccule and utricle. Vestibular evoked myogenic potential (VEMP) is one of such tests which checks function of otolith organ. VEMP can be elicited by placing the electrodes on different contracted muscles and hence the names cervical VEMP and ocular VEMP. These are electromyogenic short latency electrical impulses recorded using high acoustic stimuli. It is a proposed reliable test of saccular or inferior vestibular nerve function. In human, a vestibulo-ocular reflex stabilizes the visible world upon the retina during head and body movements. The cervical vestibular evoked myogenic potential (cVEMP) assesses the descending vestibular pathway as ipsilateral sacculocollic reflex; [1] reported the ocular vestibular evoked myogenic potential (oVEMP) to evaluate the ascending vestibular pathway as crossed vestibulo-ocular reflex. They also premised that binaural acoustic stimulation can elicit oVEMPs as effectively as monaural acoustic stimulation does. The former method requires more muscular effort than the later since continuous upward gazing is necessary during oVEMP testing. 

Normal cVEMP responses are characterized by biphasic (positive-negative) waves. The first peak is denoted as p1 followed by n1. Peaks in cVEMP are denoted with the small letter to discriminate the waveform from the neurally evoked responses [2]. oVEMP is characterized by biphasic (negative-positive) waves. The amplitude of the waveforms is mostly large and keeps varying from few microvolts to 100 microvolts, depending on the contraction of the muscle tension (with upward gazing) and stimulus intensity [3].

Most commonly in clinics, vestibular evoked myogenic potential testing is used to assess sacculocollic function in cases of vestibular neuritis, endolymphatic hydrops, superior canal dehiscence syndrome, acoustic neuroma, auditory neuropathy, and some neurodegenerative diseases [4–6]. A study on 30 noise induced hearing loss (NIHL) subjects indicated prolonged VEMP latencies and reduced peak to peak amplitude, which concluded the high possibility of vestibular dysfunction, specially the saccular pathway, in individuals with NIHL [7].

The VEMP can be tested by using different test stimuli. Click and tone burst stimuli have often been used in research on cVEMP and so are the amplitude modulated tones. Five hundred Hz short duration tone burst stimuli are most commonly used, as the animal studies revealed vestibular afferent nerve fibres to be more sensitive to the low frequencies [8, 9]. Larger VEMP amplitudes with 500 Hz tone bursts than with 1000 Hz and 2000 Hz tone bursts were observed [10]. Short latency was observed for click evoked cVEMP as compared to short duration tone burst stimuli [9, 11]. These authors reported a higher response rate for the click stimuli than the short duration tone burst stimuli. However, higher response rates were seen for the click stimulus [12]. At the same time, no difference between the two stimuli with regards to response latency or amplitude was obtained [13]. In addition, higher amplitude responses were seen over a range of modulation frequencies (5, 39, 59, 78, 98, and 122 Hz), and specifically the 39 Hz modulation frequency yielded the largest amplitude response [14]. 

Information related to the effect of different stimuli in evoking cVEMP response is vast. But there is a dearth of knowledge on the effect of different stimuli on oVEMP response and hence the present study was carried out. The aim of this study was to obtain oVEMP responses in normal hearing subjects using different test stimuli like click, 500 Hz short duration tone bursts, and amplitude modulated tones.

## 2. Materials and Method

To accomplish the objective of the study, a group of 60 healthy subjects aged 18 to 40 (mean age of 28.05 years) years were recruited. The group consisted of subjects with normal hearing sensitivity within 15 dBHL across 250 Hz to 8000 Hz for air conduction (AC) threshold and 250 Hz to 4000 Hz for bone conduction (BC). All the subjects had “A” type tympanogram with bilateral ipsilateral and contralateral acoustic reflexes present. Volunteers with uncomfortable level for speech greater <95 dBHL were included in the study. Any history or presence of any conductive hearing component or neurological symptoms, vertigo, or giddiness was not entertained in the study. 

A calibrated diagnostic GSI 61clinical audiometer was used to track the air and bone conduction pure tone thresholds. Calibrated middle ear analyzer GSI-Tympstar was used for tympanometry and reflexometry, and oVEMP recordings were done using Intelligent Hearing System Smart EP version 3.94 (Florida, USA).

The testings were carried out in a double walled sound treated room. The ambient noise level was within the levels of ANSI (1991) [15].

The air conduction and bone conduction thresholds were obtained at frequencies 250 Hz–8 KHz and 250 Hz–4 KHz, respectively, by placing the headphones and bone vibrator on both the ears and mastoid, using a mixture of both ascending and descending methods. The procedure was taken in a sound-insulated room. All the participants had normal hearing sensitivity in both ears. Uncomfortable level for speech was obtained using ascending and descending procedures. Following this, immittance audiometry was carried out. Tympanometry and reflexometry were recorded for all participants with 226 Hz probe tone. oVEMP was recorded using click, 500 Hz short duration tone burst, and 500 Hz mixed amplitude modulated stimuli. Best cVEMP response using a frequency modulation of 39 Hz with 100% amplitude modulation and 10% of frequency modulation depth at 500 Hz short duration tone burst was obtained [14] (see Table 1).

In order to achieve low impedance, the avoidance of the electrodes capturing activity from sources of interference, for example, electromagnetic radiation, was cautioned. Electrode sites were cleaned using skin preparation nu-prep gel, and then the electrodes were placed, respectively. Noninverting electrode was placed beneath the eye over the inferior oblique muscle of the eye, inverting electrode at 1-2 cm below the active electrode over the cheek and ground electrode on forehead. Electrode impedance was kept below 5 KOhm, and interelectrode impedance was kept at 2 KOhms for all the electrodes. Subjects were made to sit comfortably in a straight upright chair and were asked to relax and maintain an upward gaze at 30 degrees during the oVEMP recording. The presence of the biphasic complex was taken as the presence of VEMP. In order to achieve a high degree of reliability and validity during the measurements, the recordings were carried out twice. The n1 and p1 latencies as well as peak to peak amplitude of n1-p1 were determined for all waveform.

Statistical analysis was performed by two experienced audiologists. Statistical analysis was carried out using SPSS software version 16.0. ANOVA and post hoc Bonferronni test were carried out to see the significant difference between the recordings using different test stimuli.

## 3. Results

**Figure 1:**
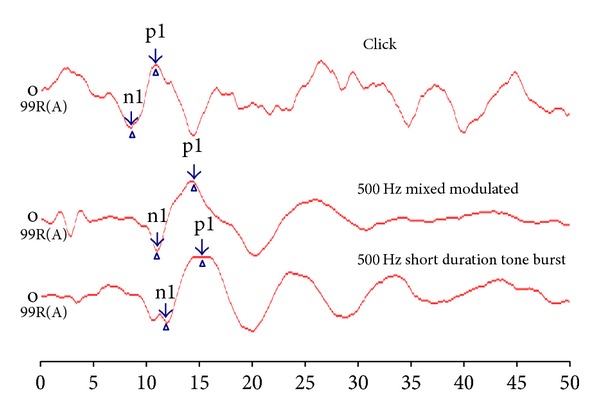
Average grand waveform for oVEMP response using click, 500 Hz mixed modulated, and 500 Hz short duration tone burst stimuli.

oVEMP was investigated using click stimulus, 500 Hz mixed modulated stimulus, and 500 Hz short duration tone burst stimulus. From Figure 1, it is evident that click oVEMP latency is better than 500 Hz short duration tone burst and 500 Hz mixed modulated stimuli in latency. However, peak-peak amplitude is better for 500 Hz mixed modulated stimulus compared to click stimulus. 

To note down the statistically significant difference, one way ANOVA and Bonferroni post hoc test were carried out. 

For the click stimulus, the latency of the n1 and p1 was significantly shorter in comparison to the 500 Hz short duration tone burst and 500 Hz mixed modulated stimuli.

**Figure 2:**
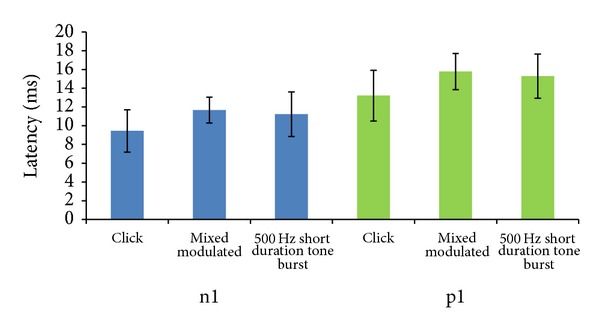
Shows mean and standard deviation of n1 and p1 latency of click, 500 Hz short duration tone burst, and 500 Hz mixed modulated signal.

From Figure 2, it is evident that mean n1 latencies of click, 500 Hz short duration tone burst, and 500 Hz mixed modulated stimuli are 9.99 ± 2.25 (SD) msec, 11.66 ± 1.37 (SD) msec, and 11.22 ± 2.37 (SD) msec, respectively. Similarly mean p1 latencies were 13.20 ± 2.69 (SD) msec, 15.77 ± 1.93 (SD) msec and 15.28 ± 2.35 (SD) msec for click, 500 Hz short duration tone burst, and 500 Hz mixed modulated signal, respectively. One way ANOVA test showed main significant effect {*F* = (2,357) = 39.23; *P* = 0.00} on n1 latency across different test stimuli. For p1 latency one way ANOVA test showed significant difference {*F* = (2,357) = 40.31; *P* = 0.00} across the test stimuli. Bonferroni analysis showed, there was better n1 and p1 latencies for click as compared to 500 Hz short duration tone burst and 500 Hz mixed modulated stimulus. When the comparison was made at 500 Hz short duration tone burst to 500 Hz mixed modulated stimulus, there was no significant difference in n1 and p1 latencies.

**Figure 3:**
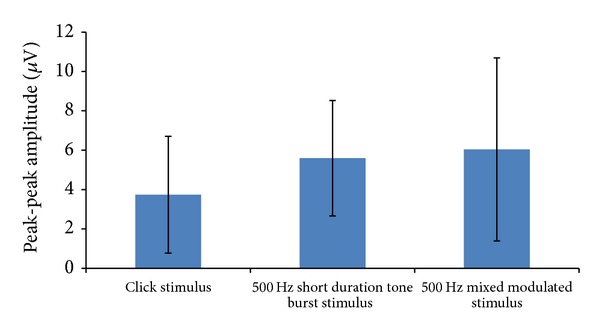
Shows mean and standard deviation of peak to peak amplitude across different test stimuli.

Peak to peak amplitude of oVEMP was recorded using different test stimuli. In Figure 3, mean peak-peak amplitude and standard deviation are shown across different test stimuli. Using click stimulus mean peak-peak amplitude was 3.74 ± 2.96 (SD) *μ*V; however, 500 Hz short duration tone burst and 500 Hz mixed modulated stimuli had 5.58 ± 2.93 (SD) *μ*V and 6.03 ± 4.64 (SD) *μ*V amplitudes, respectively.

One way ANOVA test showed main significant effect {*F* = (2,357) = 13.67; *P* = 0.00} on peak-peak amplitude across the different test stimuli. Bonferroni analysis showed no significant difference (*P* > 0.05) in peak-peak amplitude between 500 Hz short duration tone burst and 500 Hz mixed modulated stimuli. Test results revealed statically significant difference in peak to peak amplitude between click and 500 Hz stimuli (tone burst and mixed modulated signal).

## 4. Discussion

Results of the present study exhibited click to have significantly better n1 and p1 latencies as compared to 500 Hz short duration tone burst and 500 Hz mixed modulated stimuli. There was no difference in peak to peak amplitude between 500 Hz short duration tone burst stimulus and 500 Hz mixed modulated stimulus. However, peak-peak amplitude showed significant reduction in click stimulation as compared to 500 Hz short duration tone burst and 500 Hz mixed modulated stimuli. 

Similar to the present findings, [16, 17] reported significantly better n1 and p1 latencies for click compared to 500 Hz short duration tone burst stimuli. Peak-peak amplitude was significantly high for 500 Hz short duration tone burst when compared with click stimuli. Similar findings have been reported by [9, 11] using cVEMP response. 

These longer latencies may be attributed to different excitation patterns of vestibular neurons when exposed to short duration tone burst stimulus or 500 Hz mixed modulated stimulus. It has been reported that primary vestibular neurons respond to one short duration tone burst or 500 Hz mixed modulated stimulus by double or triple firing. Hence, the longer latency associated with 500 Hz short duration tone burst stimulus and the 500 Hz mixed modulated stimulus may be due to the influence of second or third electrical impulse “spikes”.

Peak-peak amplitude was observed higher for 500 Hz stimuli as compared to click and may be attributed to the difference in the energy of the stimulus spectrum; it is the primary reason why the 500 Hz short duration tone burst stimuli produced oVEMPs more effectively than the clicks. The difference of stimuli in sound frequency of 500 Hz short duration tone burst stimuli from that of click stimulus, dominantly containing higher frequencies. Reduced peak to peak amplitude may be also due to its lower mechanical energy of click, especially the energy from 500 Hz to 1000 Hz. In addition, the amplitude differences between VEMP elicited by click versus short duration tone burst and 500 Hz mixed modulated stimuli may be due to differences in the stimulus spectrum level. When comparisons were made at an equal peak to equal intensity levels, the click stimulus had a lower stimulus spectrum level than the short duration tone burst stimulus and the 500 Hz mixed modulated stimulus, due to its wider bandwidth [12].

Hence, present study suggests 500 Hz short duration tone burst to elicit best oVEMP responses as it was present in 100% of healthy subjects at higher intensity, and also it had higher peak-peak amplitude as compared to any other frequencies. However, the use of click stimulus is not recommended as it evoked less amplitude and poor wave morphology. 

## 5. Conclusions

With the results of this study, it is concluded that 500 Hz stimuli may be preferable when assessing the presence or absence of VEMP responses, as larger amplitude response facilitates peaks with good morphology. 

## Figures and Tables

**Table 1:** Parameters used to record oVEMP responses.

Parameters	Settings
Analysis time	100 msec
Filter settings	High pass: 10 Hz Low pass: 1500 Hz
Gain	5000
Stimulus type	500 Hz tone bust (8 msec). 500 Hz mixed modulated (8 msec) Click (0.1 msec).
Rate	5.1 m/sec
Polarity	Rarefaction
Intensity	99 dBnHL
Total number of sweeps	250
Transducer	ER 3A insert earphone
